# Using Google web search to analyze and evaluate the application of ChatGPT in femoroacetabular impingement syndrome

**DOI:** 10.3389/fpubh.2024.1412063

**Published:** 2024-05-31

**Authors:** Yifan Chen, Shengqun Zhang, Ning Tang, Daniel M. George, Tianlong Huang, JinPing Tang

**Affiliations:** ^1^Orthopaedic Department, The Second Xiangya Hospital of Central South University, Changsha, China; ^2^Orthopaedic Department, The Third Xiangya Hospital of Central South University, Changsha, China; ^3^Royal Adelaide Hospital, Adelaide, SA, Australia; ^4^Department of Orthopaedics, The Third People's Hospital of Chenzhou, Chenzhou, Hunan, China

**Keywords:** ChatGPT, machine learning, femoroacetabular impingement syndrome (FAI), large language model, Google

## Abstract

**Background:**

Chat Generative Pre-trained Transformer (ChatGPT) is a new machine learning tool that allows patients to access health information online, specifically compared to Google, the most commonly used search engine in the United States. Patients can use ChatGPT to better understand medical issues. This study compared the two search engines based on: (i) frequently asked questions (FAQs) about Femoroacetabular Impingement Syndrome (FAI), (ii) the corresponding answers to these FAQs, and (iii) the most FAQs yielding a numerical response.

**Purpose:**

To assess the suitability of ChatGPT as an online health information resource for patients by replicating their internet searches.

**Study design:**

Cross-sectional study.

**Methods:**

The same keywords were used to search the 10 most common questions about FAI on both Google and ChatGPT. The responses from both search engines were recorded and analyzed.

**Results:**

Of the 20 questions, 8 (40%) were similar. Among the 10 questions searched on Google, 7 were provided by a medical practice. For numerical questions, there was a notable difference in answers between Google and ChatGPT for 3 out of the top 5 most common questions (60%). Expert evaluation indicated that 67.5% of experts were satisfied or highly satisfied with the accuracy of ChatGPT’s descriptions of both conservative and surgical treatment options for FAI. Additionally, 62.5% of experts were satisfied or highly satisfied with the safety of the information provided. Regarding the etiology of FAI, including cam and pincer impingements, 52.5% of experts expressed satisfaction or high satisfaction with ChatGPT’s explanations. Overall, 62.5% of experts affirmed that ChatGPT could serve effectively as a reliable medical resource for initial information retrieval.

**Conclusion:**

This study confirms that ChatGPT, despite being a new tool, shows significant potential as a supplementary resource for health information on FAI. Expert evaluations commend its capacity to provide accurate and comprehensive responses, valued by medical professionals for relevance and safety. Nonetheless, continuous improvements in its medical content’s depth and precision are recommended for ongoing reliability. While ChatGPT offers a promising alternative to traditional search engines, meticulous validation is imperative before it can be fully embraced as a trusted medical resource.

## Background

Computer technology is rapidly advancing, paving the way for the use of artificial intelligence in medicine. Specifically, machine learning (ML), a subset of artificial intelligence, allows computer algorithms to learn from experience using large datasets without explicit programming. The potential benefits of machine learning include automating repetitive tasks, enhancing treatment decisions, predicting adverse outcomes, and streamlining perioperative healthcare management (1). In the field of orthopedics, ML has been used for multiple purposes. These include detecting mechanical loosening in hip prostheses (2), diagnosing meniscus tears (3), grading osteoarthritis (4, 5), diagnosing bone tumors, and identifying fractures (6–12). As machine learning methods continue to advance and larger datasets become more accessible, there is a growing interest in the applications of machine learning (13). OpenAI (San Francisco, California) released ChatGPT on their official website on November 30th, 2022. They described it as a sibling model to Instruct GPT that is specifically trained to understand instructions in prompts and provide detailed responses. This advanced language model has sparked a surge of interest in machine learning due to its ability to engage in complex conversations and provide information on a wide range of topics (14). In an evolving healthcare model that prioritizes consumer needs, patients now have unprecedented access to information. This includes the use of ChatGPT to gain a better understanding of medical issues. A recent study revealed that 89% of American citizens search their health symptoms on Google before scheduling a doctor’s appointment (15). The quality of online health information remains problematic, and consumers’ evaluation of such information is often subjective and misleading (16–19).

Femoroacetabular impingement (FAI) is a well-known clinical syndrome deriving from morphologic alterations of the acetabulum or femur and is a common cause of hip pain and limited hip range of motion. With the advancement of imaging technology and improved understanding of FAI, diagnosis and evaluation of this condition have become more accurate. The use of advanced imaging techniques like Magnetic Resonance Imaging (MRI) and hip arthroscopy has provided researchers with a deeper understanding of the pathogenesis, prevention, and treatment methods for FAI. Collaboration among multiple disciplines has also contributed to progress in FAI research, as professionals from various fields work together to comprehensively study FAI from different perspectives. As a result, FAI has emerged as a current research are of interest (20).

ChatGPT has already been explored in the field of orthopedics and Total Joint Arthroplasty (21). However, it has not yet been explored in the field of Femoroacetabular Impingement (FAI). Since FAI patients are often younger and more accustomed to using emerging scientific technologies like ChatGPT, we have decided to explore its appropriate and inappropriate use for collecting online health information from patients in the FAI domain. In order to assess ChatGPT in this regard, we aimed to compare it to a Google web search (Menlo Park, California), which implements well known deep learning algorithms, RankBrain, and Bidirectional Encoder Representations from Transformers (BERT), a natural language processing system that identifies search patterns quite accurately (22, 23). Google is the most widely used search engine in the United States and is also the preferred search engine for patients seeking information on orthopedic diagnosis and treatment (24). These algorithms offer a comprehensive list of frequently asked questions (FAQs) and provide links to websites that address each relevant question. Research has shown that up to 65% of patients considering elective orthopedic surgery use the internet: however, specific information they seek remains largely undefined. Additionally, the quality and readability of the available information are often poor quality (25, 26).

This study aims to evaluate the effectiveness of ChatGPT as an online health information resource for patients, specifically in the context of femoroacetabular impingement syndrome (FAI). ChatGPT, introduced in 2022 as a novel machine learning tool, is designed to provide fluent conversational responses. This study compares the capabilities of ChatGPT with those of Google Web Search, the most prevalent search engine in the United States, in three key aspects:

- Identifying and replicating the most common questions regarding FAI, with attention to the types of questions and topics covered,
- Analyzing the responses provided to these frequently asked questions, and.
- Evaluating the accuracy and relevance of numerical responses in the FAQs.The underlying hypothesis of this research is that both Google Web Search and ChatGPT will demonstrate comparable proficiency in addressing these aspects, irrespective of the specificity of the question type or topic.

## Methods

On February 6th, 2024, we conducted a series of searches on google.com using the latest version of Google Chrome browser (Version 119.0.6045.160, Menlo Park, California). The primary aim of these searches was to minimize the influence of personalized search algorithms (27). To achieve this, we implemented a ‘clean browser installation’—a process which entailed removing browsing history, cookies, website data, cached images, and files, as well as filtering out sponsored websites and advertisements. This setup was essential to ensure that the search results were not skewed by previous browsing behavior or targeted advertising. The specific search term employed was ‘femoroacetabular impingement syndrome’. Through this approach, we aimed to capture a more objective snapshot of the information available to a typical user querying this condition. This search term is entered separately to extract the top 10 frequently asked questions along with their respective sources from relevant websites. These questions are obtained from the “People also ask” section in Google web search by clicking on individual questions to reveal additional related queries. Inclusion criteria encompass any questions pertaining to “hip impingement syndrome, femoroacetabular impingement syndrome, hip impingement, hip collision, Femoro-Acetabular Impingement, Impingement, Femoracetabular.” Exclusion criteria involve eliminating duplicate questions and those unrelated to femoroacetabular impingement syndrome (for example: “What happens after gluteal muscle contracture surgery?”).

ChatGPT is a chatbot developed by OpenAI and released in November 2022. It is a freely accessible and replicable language model designed for various natural language processing tasks, including text generation and language translation. GPT-4, the successor to GPT-3.5, significantly expands upon its predecessor’s capabilities, featuring an even more sophisticated architecture with approximately 175 billion parameters. While the exact number of parameters for GPT-4 has not been publicly disclosed, it is known for its enhanced understanding and generation of natural language, improved contextual awareness, and the ability to generate more accurate and nuanced responses. GPT-4 represents a substantial leap forward in AI language models, pushing the boundaries of machine learning and natural language processing technologies (28). ChatGPT uses a Transformer model network that incorporates the concept of “attention” to generate responses based on word associations. This enables the deep learning model to assign varying levels of importance to different parts of a sentence. As a result, it produces realistic and human-like responses, which can be particularly useful in static environments or situations where the question/response remains unchanged over time. For instance, consider the non-fluctuating problem of asking how to say hello in Spanish. To demonstrate ChatGPT’s capabilities, we inputted the following statement: “Perform a google search with the search term ‘Femoroacetabular Impingement Syndrome’ and record the 10 most frequently asked questions related to this topic.” We then recorded the top 10 questions and their corresponding answers for each output.

To compare the ability of ChatGPT and Google in providing information to patients, we conducted a study using the search term “Femoroacetabular Impingement Syndrome” for both platforms. We identified the top 10 most common questions and the top 5 most frequently asked numerical questions (Tables 1–3) from Google web search results. These questions were then inputted into ChatGPT to record question-answer pairs. This analysis helps us understand the commonly asked questions by patients and allows us to assess how well ChatGPT and Google provide relevant information. It is worth noting that although Google is widely used by patients to obtain online health information, the quality of information obtained through Google searches varies. Among the information provided by Google, sources like PubMed may offer more reliable answers (15, 23). PubMed can be a valuable resource for future comparisons. This study aims to replicate patients’ internet searches and evaluate the suitability of ChatGPT. Compared to Google Web Search, ChatGPT offers fluent conversational responses to queries, making it an important online health information resource for patients.

**Table 1:** Top 10 most popular questions for FAI according to Google web search.

1.How do you fix femoroacetabular impingement?
2.What causes hip impingement syndrome?
3.Can hip impingement be cured?
4.What is hip femoroacetabular impingement syndrome?
5.What is the fastest way to fix hip impingement?
6.Can you fix hip impingement without surgery?
7.What happens if hip impingement is left untreated?
8.ls cycling bad for hip impingement?
9.Can you live with hip impingement?
10.Does walking make hip impingement worse?

**Table 2:** Top 10 most popular questions for FAI according to ChatGPT.

1.What is Femoroacetabular Impingement Syndrome (FAIS)?(Notion)
2.What are the symptoms of FAI?(Technical Details)
3.What causes FAIS?(Technical Details)
4.How is FAIS diagnosed?(Technical Details)
5.What are the treatment options for FAIS?(Treatment Guidelines and Options)
6.Can FAIS be managed without surgery?(Treatment Guidelines and Options)
7.What is the recovery time after FAIS surgery?(Timeline of Recovery)
8.Are there any exercises or physical therapy for FAIS?(Treatment Guidelines and Options)
9.Can FAIS lead to hip osteoarthritis?(Potential Consequences and Complications)
10.Can FAIS affect both hips?(Potential Consequences and Complications)

**Table 3:** Top 5 questions and answer comparison of search engines for numerical questions about femoroacetabular impingement syndrome.

Question	Google	ChatGPT
How many people have femoroacetabular impingement?	0.0544% (Pubmed)	10 to 15%
How long does femoroacetabular impingement last?	4-6 m	Vary with each individual
What is the common age for FAl?	25–50	20–30
What is the success rate of FAl hip surgery?	85–90%	70–90%
How long is FAl recovery?	4-6 m	6-12w

The questions retrieved in our research are classified into problem themes: fact, policy, and value. This classification is based on previous literature that utilized the modified Rothwell system (29, 30). These issues are divided into 9 themes related to femoroacetabular impingement syndrome: specific activities, rehabilitation timeline, concepts, technical details, costs, treatment guidelines and options, potential consequences and complications, curability and quality of life, as well as patient preferences and decision-making. The website is categorized into the following groups: commercial category, academic category, medical practices (or private clinics), individual surgeons specializing in a single field, government institutions, and social media (Table 4) (31, 32). The Cohen’s kappa coefficient is utilized to assess the inter-rater reliability of website classification. We used IBM SPSS Statistics (Version R24.0.0.0, Armonk, New York) to conduct statistical analysis on website categorization and problem classification. In the case of website classification, the kappa value for inter-rater reliability is 1 (*p* < 0.01). For problem classification, the kappa value for inter-rater reliability is 0.702 (*p* < 0.01).

**Table 4:** Question topics and website categorization by Rothwell’s classification.

Question topics categorized by Rothwell’s classification	
*Fact*	Asks whether something is true and to what extent, objective information
Specific activities	Ability to perform a specific activity or action
Timeline of recovery	Length of time for recovery milestones
Notion	Conceptual issues related to FAI
Technical details	Detail problems in diagnosis and treatment
Cost	Cost of surgery and/or rehabilitationSurgical procedure and anesthesia, includes specifics about implants
*Policy*	Asks whether a specific course of action should be taken to solve a problem
Treatment guidelines and options	Questions related to treatment guidelines and options
Potential consequences and complications	Questions that involve potential consequences, complications, or risks associated with treatment
*Value*	Asks for evaluation of an idea, object, or event
Curability and quality of life	Issues related to the ability of the disease to be treated and the quality of life after treatment
Patient preferences and decision-making	Problems related to the patient’s subjective approach to treatment
*Website categorization*	
Commercial	Universities, academic medical centers, or academic societies
Academic	Universities, academic medical centers, or academic societies
Medical practice	Local hospitals or medical groups without clear academic affiliation
Single surgeon practice	Personal websites maintained by individual surgeons
Government	Websites maintained by a national government
Social media	Blog, internet forums, support groups, and nonmedical organizations designed for information and video sharing

To rigorously assess the accuracy and applicability of information sourced from ChatGPT, we enlisted 40 orthopedic specialists with extensive experience in hip arthroscopy. Their evaluations provide critical insights into the practical utility of ChatGPT responses in clinical settings.

The expert panel was diverse, consisting of 32.5% chief physicians, 57.5% associate chief physicians, and 10% attending physicians. Their annual surgical volume varied, with 30% performing fewer than 10 hip arthroscopies per year and 12.5% performing over 100, indicating a broad range of clinical activity.

Experts were asked to assess the quality of ChatGPT’s answers to questions related to femoroacetabular impingement syndrome (FAI) based on accuracy, completeness, relevance, and understandability. The evaluation used a five-point scale ranging from very satisfied to very dissatisfied.

## Results

### Most popular questions for Google web search and ChatGPT

When searching for “Femoroacetabular Impingement Syndrome” on both Google web search and ChatGPT search, 8 out of 20 questions (40%) were found to be similar. The most common theme category was Fact, with 5 out of 10 questions in both Google web search and ChatGPT falling into this category. Based on themes, the more common subcategories were Specific Activities (3 out of 10 questions) for Google web search, and Timeline of Recovery (3 out of 10 questions) as well as Technical Details (3 out of 10 questions) for ChatGPT (Tables 1, 2).

### Answers to the most popular questions

In Google web search, Medical Practice is the most common source of answers for the top 10 questions, accounting for 7 out of 10. There are a total of 15 answers, including those for the top 5 numerical questions. The specific questions asked are as follows: (1) “How many people have femoroacetabular impingement?,” (2) “How long does femoroacetabular impingement last?,” (3) “How long is FAI recovery?,” (4) “Can hip impingement be cured?,” (5) “Is cycling bad for hip impingement?,” (6) “Can you live with hip impingement?,” (7) “Does walking make hip impingement worse?” (Tables 3–6). It should be noted that Google and ChatGPT provide noticeably different suggestions.

### Most popular questions for discrete questions

Medical Practice provided answers for 7 out of the top 10 questions searched on Google. Among the top 5 most frequently asked numerical questions, there were 3 (60%) that had different answers between Google search and ChatGPT (Table 3). The question with the largest discrepancy in data was “How many people have femoroacetabular impingement?.” For the top 10 most common questions about femoroacetabular impingement, there were different responses for 4 out of 10 questions (40%) (Tables 5, 6). Only two replies obtained from Google search for these 10 questions (20%) used sources from academic websites (Tables 5, 6).

**Table 5:** Answers to most popular questions for FAI.

Question	Google	ChatGPT
1.What is the fastest way to fix hip impingement?	Hip impingement treatments resting the affected hip. Modifying your activities to avoid moving the joint in a way that causes pain. Exercising as recommended by your doctor or physical therapist to strengthen the muscles that support the hip *(Commercial)*	The treatment for femoroacetabular impingement (FAI), depends on several factors, including the severity of symptoms, the extent of joint damage, and the individual’s overall health. While there is no “fast” way to fix hip impingement, here are some steps that can help manage hip impingement: 1.Seek Professional Evaluation; 2.Conservative Treatment; 3.Surgical Intervention: Arthroscopic Surgery; Open Hip Surgery. It’s important to note that the treatment approach and timeline will vary for each individual.Working closely with your healthcare team will help guide you through the treatment process and optimize your outcomes.
2.What happens if hip impingement is left untreated?	Over time, impingement of the hip may tear the labrum — a fibrous ring of cartilage surrounding the hip socket. When the labrum is torn, it can cause hip pain. Hip impingement also may damage the protective cartilage that covers the ends of the bones in the joint, called articular cartilage *(Medical practice)*	If left untreated femoroacetabular impingement (FAI), can lead to various complications and long-term consequences. Here are some potential outcomes: 1. Persistent Pain; 2.Labral Tears; 3. Cartilage Damage; 4. Hip Joint Degeneration; 5. Functional Limitations; 6. Secondary Hip Conditions.
3.ls cycling bad for hip impingement?	Factors that increase the risk of developing FAI include: Repetitive activities causing hip flexion (cycling) A low saddle height causing excessive hip flexion. Anatomical abnormalities *(Medical practice)*	Cycling can be a beneficial form of exercise for individuals with hip impingement. However, it is important to consider certain factors and make modifications to ensure that cycling does not exacerbate hip impingement symptoms. Here are some considerations: 1. Bike Fit; 2.Cadence and Resistance; 3. Posture and Techniqu; 4. Modify Position: Experiment with different handlebar and saddle positions. 5.Manage Intensity and Duration: Start with shorter and less intense rides, gradually increasing the duration and intensity. 6.Cross-training and Strengthening: Incorporate cross-training activities, such as stretching and strengthening exercise.
4.Can you live with hip impingement?	If diagnosed with femoroacetabular or hip impingement, there are both nonsurgical and surgical treatment plans. No impingement will heal on its own. If left untreated, a hip impingement may lead to osteoarthritis in the hip joint *(Medical practice)*	Yes, it is possible to live with femoroacetabular impingement (FAI). The impact of hip impingement on an individual’s daily life can vary depending on the severity of symptoms, the extent of joint damage, and the individual’s overall health and activity level. Some individuals with mild impingement may have minimal symptoms and can continue to lead active lives without requiring extensive treatment. However, it’s important to note that hip impingement can potentially lead to long-term consequences if left untreated. Untreated impingement can result in persistent pain, labral tears, cartilage damage, and joint degeneration over time.
5.Does walking make hip impingement worse?	So, walking and any attempt to become physically active may cause great difficulty and worsening of pain. Patients with severe hip arthritis may even experience pain even after stopping physical activity. For hip arthritis so severe that walking becomes an impossibility, please consult an orthopedic surgeon *(Medical practice)*	Walking is generally considered a low-impact activity that can be beneficial for individuals with femoroacetabular impingement, as long as it is performed with proper technique and consideration for individual symptoms. Here are some factors to consider: 1. Symptom Management; 2. Gait Mechanics; 3. Surface and Terrain; 4. Distance and Intensity; 5. Supportive Footwear.

**Table 6:** Answers to most popular questions for FAI.

Question	Google	ChatGPT
1.How do you fix femoroacetabular impingement?	Femoroacetabular impingement (FAI), also called hip impingement, is a condition where the hip joint is not shaped normally. This causes the bones to painfully rub together. This condition can be treated with corticosteroids, nonsteroidal anti-inflammatory drugs (NSAIDs), physical therapy, rest and surgery *(Medical practice)*	Femoroacetabular impingement (FAI) can be managed and treated through both conservative (non-surgical) and surgical approaches. Here are some common treatment options for femoroacetabular impingement: Conservative Treatment(Rest and activity modification and Physical therapy, Nonsteroidal anti-inflammatory drugs,Injections) and Surgical Treatment(Hip arthroscopy, Open hip surgery,Osteotomy).The choice of treatment depends on various factors, such as the patient’s age, activity level, severity of symptoms, and any associated hip conditions.
2.What causes hip impingement syndrome?	It is caused when the femoral head (the ball-shaped bone at the top of the femur or thigh bone) and the acetabulum (the socket in the pelvis into which the femoral head fits) do not fit together properly. This is often the result of extra bone in the hip, though it can also be caused by bone deformity *(Academic)*	Hip impingement syndrome, also known as femoroacetabular impingement (FAI), There are two main types of FAI: cam impingement and pincer impingement, and in some cases, both types can be present simultaneously. Here’s a breakdown of the causes for each type of impingement:Cam Impingement (Excess bone growth,Abnormal hip joint mechanics)and Pincer Impingement (Overcoverage of the acetabulum, Abnormal hip socket alignment).
3.What is hip femoroacetabular impingement syndrome?	Femoroacetabular impingement (FAI) is a condition in which extra bone grows along one or both of the bones that form the hip joint — giving the bones an irregular shape. Because they do not fit together perfectly, the bones rub against each other during movement *(Academic)*	Femoroacetabular impingement syndrome is a condition characterized by abnormal contact between the femoral hea and the acetabulum in the hip joint. FAI syndrome can be categorized into two main types: 1.Cam Impingement; 2.Pincer Impingement. The abnormal contact can cause inflammation, pain, labral tears and eventual cartilage damage or hip osteoarthritis if left untreated. Symptoms may include hip pain, stiffness, clicking or catching sensations, limited range of motion, and discomfort during activities like walking, sitting, or sports.
4.Can you fix hip impingement without surgery?	Non-surgical treatment should always be considered first when treating impingement. This condition can often be resolved with rest, modifying activity behavior to adapt to change in hip structure, physical therapy input and/or appropriate painkillers *(Medical practice)*	In some cases, femoroacetabular impingement (FAI), can be managed without surgery, especially if the symptoms are not severe. Here are some non-surgical options for managing hip impingement: 1. Physical Therapy; 2. Activity Modification:; 3. Medications: Nonsteroidal anti-inflammatory drugs (NSAIDs); 4. Injections: Corticosteroid injections into the hip joint; 5. Assistive Devices. If conservative treatments do not sufficiently alleviate symptoms or if there is significant joint damage, surgical intervention may be recommended.
5.Can hip impingement be cured?	However, most patients will make a full recovery in four to 6 months. Many instances of FAI can be adequately managed with a personalized approach to conservative care treatment. However, arthroscopic procedures are becoming more popular with competitive athletes and active adults *(Medical practice)*	Femoroacetabular impingement (FAI), is a structural abnormality in the hip joint. While it cannot be completely “cured” in the sense of reversing the underlying anatomy, it can often be effectively managed and treated. The goal of treatment is to reduce symptoms, improve hip function, and prevent further damage to the joint. Here are some key points regarding the management of hip impingement: Conservative Management may include: Rest and activity modification; Physical therapy; Medications: Nonsteroidal anti-inflammatory drugs (NSAIDs); Injections; Assistive devices. Surgical Intervention include: Hip arthroscopy; Open hip surgery; Osteotomy. It’s important to note that early detection and timely intervention can lead to better outcomes.

### Expert evaluation results

#### Expert evaluation insights on ChatGPT’s handling of femoroacetabular impingement syndrome (FAI)

##### Treatment methods

A substantial 67.5% of experts reported satisfaction or high satisfaction with the accuracy of ChatGPT’s descriptions of both conservative and surgical treatment options for FAI. Furthermore, 62.5% of experts indicated their satisfaction or high satisfaction with the safety of the information, suggesting a significant trust in ChatGPT’s responses to safely guide patients. This result confirms the strong approval of the content’s relevance and practicality, with a similar percentage also acknowledging that the information was comprehensively suitable for patient use.

Etiology of FAI: In terms of explaining the etiology of FAI, specifically cam and pincer impingements, 52.5% of experts expressed satisfaction or very satisfaction with ChatGPT’s explanations. This satisfaction rate emphasizes ChatGPT’s ability to handle complex medical topics and translate intricate medical knowledge into comprehensible information.

##### Overall potential

When assessing the potential of ChatGPT to act as a reliable medical resource for initial information retrieval, 62.5% of experts confirmed that ChatGPT could effectively fulfill this role. This endorsement underscores ChatGPT’s adaptability in medical settings and solidifies its position as a valuable educational tool in healthcare.

##### Reliability analysis

The internal consistency of the responses, as measured by Cronbach’s alpha, was notably high (α = 0.908). This high level indicates a reliable assessment across different evaluators, further validating the dependability of the feedback received.

## Discussion

Publi cations on machine learning in the field of orthopedics have increased tenfold in the past 20 years (33). The increasing availability of information and modern data in today’s value-based era of medicine may be the reason for this (34). ChatGPT is a machine learning tool that has gained attention for its accessibility, user-friendliness, engaging interface, and limitless possibilities. Femoroacetabular impingement (FAI), also known as hip impingement syndrome or hip joint impingement syndrome, is a condition affecting the hip joint. It involves symptoms caused by friction, collision, or compression between the femoral head and acetabulum. This syndrome usually results from morphological changes in the acetabulum or femur and commonly leads to hip pain and limited range of motion (20). Femoroacetabular impingement syndrome is a common condition that significantly affects patients’ quality of life, the overall incidence of FAI diagnosis was 54.4 per 100,000 person-years (35). It has been extensively studied for many years due to its prevalence, particularly among young individuals who make up a significant portion of ChatGPT users. Young people are generally tech-savvy and comfortable with using artificial intelligence (AI) assistants and chatbots like ChatGPT for various purposes such as entertainment, learning, information-seeking, and problem-solving. Considering the overlap between the user base of ChatGPT and the population affected by FAI, it is important to note that people’s use of ChatGPT to search for information about FAI can contribute to both the development of ChatGPT and influence their healthcare decision-making process accordingly.

ChatGPT differs from traditional search engines like Google in its learning mechanisms. Firstly, ChatGPT utilizes deep learning techniques, specifically the Transformer model, trained on large-scale corporate data. In contrast, traditional search engines primarily rely on information retrieval techniques such as keyword matching and ranking algorithms. Secondly, ChatGPT is a conversational language model that allows real-time interaction and dialogue. It can understand user questions, responses, and instructions while generating coherent and personalized replies. Traditional search engines require users to input keywords and return web page links and search results related to those keywords. Furthermore, ChatGPT can comprehend and remember previous conversation history while considering context when answering questions. This enables it to provide more coherent and personalized responses and always suggests following the guidance of professionals at the end of each answer, demonstrating a strong sense of rigor and risk avoidance ability. Traditional search engines mainly rely on phrase matching without taking into account a user’s prior queries or conversations. Lastly, ChatGPT can answer complex questions through inference and text generation while making educated guesses based on contextual cues. It possesses a certain level of creativity and flexibility in generating new textual content. Conversely, traditional search engines heavily depend on pre-established indexes and webpage content without actively reasoning or generating new text themselves.

The purpose of this study is to assess the suitability of patients’ internet searches as a means of accessing online health information, specifically comparing ChatGPT to Google web search. Our findings reveal notable distinctions between Google web search and ChatGPT in terms of the most frequently asked questions and corresponding answers about FAI. Furthermore, we observed that while Google web search relies on Medical Practice as a primary source for answer references, ChatGPT does not directly access the internet or specific websites like Google does. Consequently, we were unable to obtain specific website sources for ChatGPT.

There are a few limitations to consider in this study. Firstly, the number of questions included is limited, which may affect the generalizability of the findings. However, despite this limitation, the study provides valuable insights into the potential of this emerging machine learning (ML) tool due to its novelty and dedicated research efforts. It’s important to note that ChatGPT is a deep learning tool trained on text data from 2021 and does not have internet access like Google Web Search. While ChatGPT utilizes various web text sources during training such as web pages, books, papers, and publicly available content, it cannot retrieve specific information from websites like Google Web Search can. The Google Web Search used in this study was conducted On February 6th, 2024. Differences in databases between both searches could explain any discrepancies found in common questions and answers obtained during our research. Additionally, while ChatGPT remains unchanged throughout the study period, Google’s algorithm may have undergone changes that cannot be replicated. It’s worth noting that medical care standards and treatment methods evolve over time. This evolution might lead to inconsistencies with ChatGPT’s ability to generate accurate human-like responses since accuracy relies on a training set that reflects current issues and addresses non-stationary environments or non-volatile problems (where questions/responses remain consistent over time). An example of a non-volatile problem would be “What is the normal human body temperature?” In this analysis, we chose to compare ChatGPT with Google Web Search because it is widely used as a search engine in the United States (23), but if you use other search engines like Bing (Redmond, Washington), DuckDuckGo (Paoli, Pennsylvania), or Yahoo (Sunnyvale, California), the results should be similar. A study was conducted to compare the performance of natural language search engines and keyword-based search engines in accurately retrieving answers to natural learning queries. The author discovered that there was no significant difference in the accuracy of retrieving answers among four search engines: Google, Ask.com (Oakland, California), Hakia (New York City) and Bing (Redmond, Washington) (36). However, to the author’s knowledge, Google web search is the only search engine that provides additional FAQs when specific questions are clicked on. This feature sets it apart from Bing, DuckDuckGo, and Yahoo. By utilizing this functionality, the author can gather the top 10 most common questions without restricting future analysis to a limited number of inquiries. It should be noted that we have not considered any selection biases related to demographic data of Google search engine or ChatGPT users. It is widely recognized that factors such as age, gender, education level, and income can influence internet usage patterns and may also impact the utilization of ChatGPT (37, 38). Furthermore, the results obtained from ChatGPT may vary depending on whether more specific or less specific questions are inputted. However, it is important to note that this study’s main objective is to assess ChatGPT’s ability to replicate Google web search queries. To the best of the author’s knowledge, this analysis represents the first attempt to incorporate ChatGPT into a research design about FAI for evaluating its suitability in providing common questions and answers sought by patients compared to a search engine like Google. It focuses on its role in addressing patient queries rather than its application in scientific writing, commentary, and editing (39).

When searching for “Femoroacetabular Impingement Syndrome” on both Google web search and ChatGPT search, 8 out of 20 questions (40%) were found to be similar. However, it is important to note that although the questions are different, most of the queries made using each search tool are related to “facts.” Out of the 10 common questions found on Google, only 3 pertain to curability and quality of life, which ChatGPT attempts to replicate but does not appear in Google’s top 10 questions. This distinction is significant because curability and quality of life greatly influence patients’ treatment decision-making process. Further investigation may be necessary to understand why these different questions arise from these search tools. It should also be noted that due to its learning model, ChatGPT may be more sensitive to unique word choices used in queries compared to Google web search (40). ChatGPT can be effectively used to enhance patients’ online searches, providing them with valuable information that may not be readily accessible. However, it is important to note that relying solely on ChatGPT for answers may lead to open-ended and ambiguous responses. Therefore, it is recommended that patients consult their doctors for more complex and evolving questions.

Google web search and ChatGPT provide different answers to the most popular questions. ChatGPT provides longer responses to the most popular questions and often suggest consulting a doctor for further evaluation (Tables 5, 6 show shortened ChatGPT responses due to space constraints). This is how ChatGPT offers balanced and impartial information to patients. However, excessively long replies can affect readability.

Discrete questions and answers were similarly disparate between the search tools. For instance, when asking “How many people have femoroacetabular impingement?,” Google web search yields a result of 0.0544%, while ChatGPT provides a range of 10 to 15%. According to previous research, Google’s answer is more reliable, the overall incidence of FAI diagnosis was 54.4 per 100,000 person-years, and it consistently increased between 2000 and 2016 (35). These are just two out of seven different responses that offer varying answers. Such variations can impact patient decisions, considering factors like symptoms at different stages post-injury, diverse outcomes of conservative and surgical treatments, and the effects of various activities on rehabilitation. It’s worth noting that the response from Google web search is sourced from Pubmed, whereas ChatGPT relies on its pre-trained dataset. However, it is important to acknowledge that information obtained from internet sources for evaluation may be unfiltered, unreviewed or inaccurate.

After analyzing the most frequently asked questions about FAI, it was observed that the majority of inquiries revolve around conservative or non-surgical treatments. Interestingly, the cost category in Rothwell’s classification (Table 4) did not feature prominently in these popular questions. This suggests that public concerns regarding FAI primarily center on avoiding surgical intervention and understanding the potential consequences of untreated FAI. Furthermore, the relationship between FAI and physical activity is one of the most concerning topics for the public (Tables 1, 2). Understanding the relationship between physical activity and recovery in FAI is crucial for patients seeking health information online. Our research has shown that individuals who engage in high-impact sports such as soccer, dance, gymnastics, basketball, and ice hockey have a higher incidence of FAI compared to those who are not physically active. (41) Furthermore, sedentary individuals are also at a higher risk group. Femoroacetabular impingement syndrome can lead to structural abnormalities or functional impairments in the hip joint, which may affect comfort and mobility during physical activities. Engaging in appropriate exercises can be beneficial in managing and rehabilitating FAI, highlighting the importance of accessing accurate and tailored health information. This underscores the relevance of our study in evaluating ChatGPT as a potential online health information resource, offering insights into how patients with FAI might use it to seek advice on effective physical activities and recovery strategies. By understanding the information needs of FAI patients, especially those related to exercise and recovery, our study aims to assess the accuracy and utility of ChatGPT in providing relevant and reliable health information, aligning closely with the broader purpose of our research. Notably, when it comes to determining the incidence rate of FAI among populations (Table 3), Google search results and ChatGPT responses provided conflicting information. Based on previous research, the overall incidence of FAI diagnosis was 54.4 per 100,000 person-years, and it consistently increased between 2000 and 2016 (8).

Several studies have shown that patients who discuss their online health survey results during consultations experience improved doctor-patient relationships (17, 42, 43). According to a randomized controlled study, patient education websites have been found to enhance patients’ comprehension of their surgery and satisfaction with the consent process when compared to standard discussions with treating surgeons (44). In conclusion, these findings suggest that patients can benefit from using search tools and actively communicating accurate and useful information with their doctors. However, the full extent of this combination’s effectiveness is still not fully understood.

Since its inception, ChatGPT has rapidly become a popular resource for health-related information, attracting over 100 million users within just 2 months of its launch. Although Google continues to be the primary source for medical queries, used by up to 89% of American citizens (45), ChatGPT provides a more user-friendly interface. It is devoid of the frequent advertisements and commercial content that often clutter traditional search engines. Consequently, ChatGPT has emerged as a compelling alternative for patients seeking diverse health information, especially in contexts where immediate, comprehensible guidance is essential.

The expert evaluation of ChatGPT’s responses to questions concerning Femoroacetabular Impingement Syndrome (FAI) substantiates its potential as a healthcare resource. An impressive 67.5% of orthopedic specialists reported either satisfaction or high satisfaction with the accuracy of ChatGPT’s descriptions of both conservative and surgical treatment options. Furthermore, a similar proportion of experts confirmed the safety and comprehensiveness of the information provided, affirming that ChatGPT can reliably deliver valuable medical guidance to patients. While the expert feedback on ChatGPT has been predominantly positive, emphasizing its capability to effectively handle complex medical topics, it also highlights the necessity for continuous improvements. Experts have advocated for further enhancements in the depth and precision of the medical content, aiming to ensure that ChatGPT consistently provides high-quality information that adheres to professional medical standards.

In conclusion, although ChatGPT shows significant promise as a medical resource, its use should still be approached with caution. It is crucial to ensure the integrity of the information and minimize the risk of disseminating misleading content. Thus, ongoing validation and development are essential before ChatGPT can be fully integrated into medical practice as a trusted resource. The enthusiastic yet cautious reception by medical professionals suggests a future where AI tools like ChatGPT could transform patient education and preliminary medical consultation, provided that their development is consistently guided by rigorous expert feedback and stringent medical accuracy standards.

### What is known about the subject

Femoroacetabular impingement (FAI) is a well-known clinical syndrome deriving from morphologic alterations of the acetabulum or femur and is a common cause of hip pain and limited hip range of motion. Existing literature has extensively investigated FAI’s etiology, diagnostic methods, and treatment strategies, highlighting the need for accurate and timely interventions to mitigate its impact on patients’ quality of life. Nevertheless, the field of FAI-related research currently exhibits a paucity of in-depth exploration into the realm of artificial intelligence (AI) and machine learning. Specifically, comprehensive inquiries investigating the assimilation of AI technologies within this context are still in their incipient stages. This prevailing gap calls for an increased focus on unveiling the potential of AI-driven methodologies within the FAI landscape.

Computer technology is rapidly advancing, paving the way for the use of artificial intelligence in medicine. Specifically, machine learning (ML), a subset of artificial intelligence, allows computer algorithms to learn from experience using large datasets without explicit programming. In the field of orthopedics, ML has been used for multiple purposes. ChatGPT is a model that is specifically trained to understand instructions in prompts and provide detailed responses.

As such, our study addresses this gap by assessing the application of ChatGPT and Google in delivering accurate and relevant medical information about FAI. By comparing the responses provided by these platforms, our research contributes to an enhanced comprehension of the potential benefits and limitations of AI-based resources in the orthopedic domain.

### What this study adds to existing knowledge

ChatGPT has already been explored in the field of orthopedics and Total Joint Arthroplasty. However, it has not yet been explored in the field of Femoroacetabular Impingement (FAI). Given the typically younger age demographic of FAI patients, who readily embrace emerging scientific technologies like ChatGPT, coupled with the inclination of these youthful individuals to proactively seek out disease-related information online both prior to and following medical consultations, our study is designed to delve into the nuanced exploration of ChatGPT’s suitable and inappropriate applications within the realm of FAI. This inquiry aims to illuminate the judicious utilization of ChatGPT as a conduit for furnishing online health information to FAI patients, thereby contributing to a comprehensive comprehension of its utility and constraints within this specific medical context. Therefore, this study employs a Cross-Sectional Study design to investigate the application of ChatGPT and Google in the context of Femoroacetabular Impingement (FAI). This approach addresses the existing gap regarding the utilization of ChatGPT in the realm of FAI, enabling an assessment of its viability as an online health information resource for patients. By comparing the responses provided by these platforms, our research contributes to an enhanced comprehension of the potential benefits and limitations of AI-based resources in the orthopedic domain. Furthermore, our research serves as a reference for healthcare professionals in effectively engaging with patients who possess a wealth of online medical knowledge during doctor-patient communication.

## Data availability statement

The original contributions presented in the study are included in the article/Supplementary material, further inquiries can be directed to the corresponding authors.

## Author contributions

YC: Writing – original draft. SZ: Writing – original draft. NT: Methodology, Software, Supervision, Writing – original draft. DG: Data curation, Formal analysis, Resources, Visualization, Writing – original draft. TH: Writing – review & editing. JT: Writing – review & editing.
