# Supplementary material for: Using Google web search to analyze and evaluate the application of ChatGPT in femoroacetabular impingement syndrome
Source: Front Public Health. 2024 May 31;12:1412063. doi: 10.3389/fpubh.2024.1412063 (PMC11176516; doi:10.3389/fpubh.2024.1412063)
Supplement: Supplementary file 1 [file Table_1.docx]

**Supplementary materials**

# **1. specific queries we used in our research, along with sample responses from both Google and ChatGPT:**

**Question 1 : How do you fix femoroacetabular impingement?**

**Google :**

Femoroacetabular impingement (FAI), also called hip impingement, is a condition where the hip joint is not shaped normally. This causes the bones to painfully rub together. This condition can be treated with corticosteroids, nonsteroidal anti-inflammatory drugs (NSAIDs), physical therapy, rest and surgery.

**ChatGPT :**

Femoroacetabular impingement (FAI) can be managed and treated through both conservative (non-surgical) and surgical approaches. The appropriate treatment depends on the severity of symptoms, the underlying cause, and the individual's specific circumstances. Here are some common treatment options for femoroacetabular impingement:Conservative Treatment(Rest and activity modification and Physical therapy，Nonsteroidal anti-inflammatory drugs ,Injections) and Surgical Treatment(Hip arthroscopy,Open hip surgery ,Osteotomy).The choice of treatment depends on various factors, such as the patient's age, activity level, severity of symptoms, and any associated hip conditions. It is important to consult with a qualified orthopedic specialist who can evaluate your specific situation and recommend the most appropriate treatment option for you.

**Question 2 : What causes hip impingement syndrome?**

**Google :**

It is caused when the femoral head (the ball-shaped bone at the top of the femur or thigh bone) and the acetabulum (the socket in the pelvis into which the femoral head fits) don't fit together properly. This is often the result of extra bone in the hip, though it can also be caused by bone deformity.

**ChatGPT :**

Hip impingement syndrome, also known as femoroacetabular impingement (FAI), There are two main types of FAI: cam impingement and pincer impingement, and in some cases, both types can be present simultaneously. Here's a breakdown of the causes for each type of impingement:Cam Impingement(Excess bone growth,Abnormal hip joint mechanics)and Pincer Impingement(Overcoverage of the acetabulum,Abnormal hip socket alignment).Consulting with an orthopedic specialist can provide a more accurate.

**Question 3 : What is hip femoroacetabular impingement syndrome?**

**Google :**

Femoroacetabular impingement (FAI) is a condition in which extra bone grows along one or both of the bones that form the hip joint — giving the bones an irregular shape. Because they do not fit together perfectly, the bones rub against each other during movement.

**ChatGPT :**

Femoroacetabular impingement syndrome is a condition characterized by abnormal contact between the femoral hea and the acetabulum in the hip joint. FAI syndrome can be categorized into two main types: 1.Cam Impingement; 2.Pincer Impingement. The abnormal contact can cause inflammation, pain, labral tears and eventual cartilage damage or hip osteoarthritis if left untreated. Symptoms may include hip pain, stiffness, clicking or catching sensations, limited range of motion, and discomfort during activities like walking, sitting, or sports. Diagnosis typically involves a combination of medical history assessment, physical examination, imaging studies and sometimes diagnostic hip injections to confirm the source of pain. Treatment for FAI syndrome can involve conservative measures and surgical measures. It's important to consult with a qualified orthopedic specialist to evaluate your symptoms.

**Question 4 : Can you fix hip impingement without surgery?**

**Google :**

Non-surgical treatment should always be considered first when treating impingement. This condition can often be resolved with rest, modifying activity behaviour to adapt to change in hip structure, physical therapy input and/or appropriate painkillers.

**ChatGPT :**

In some cases, femoroacetabular impingement (FAI), can be managed without surgery, especially if the symptoms are not severe. Here are some non-surgical options for managing hip impingement: 1.Physical Therapy: Using exercise program to strengthen the hip muscles. 2.Activity Modification: Avoid or modify activities that exacerbate symptoms. 3.Medications: Nonsteroidal anti-inflammatory drugs (NSAIDs), may be recommended to reduce pain and inflammation. 4.Injections: Corticosteroid injections into the hip joint can provide temporary relief by reducing inflammation and pain. 5. Assistive Devices: In some cases, using crutches or a cane to offload the hip joint during weight-bearing activities can help reduce symptoms and allow the joint to heal. If conservative treatments do not sufficiently alleviate symptoms or if there is significant joint damage, surgical intervention may be recommended.

**Question 5 : Can hip impingement be cured?**

**Google :**

However, most patients will make a full recovery in four to six months. Many instances of FAI can be adequately managed with a personalized approach to conservative care treatment. However, arthroscopic procedures are becoming more popular with competitive athletes and active adults.

**ChatGPT :**

Femoroacetabular impingement (FAI), is a structural abnormality in the hip joint. While it cannot be completely "cured" in the sense of reversing the underlying anatomy, it can often be effectively managed and treated. The goal of treatment is to reduce symptoms, improve hip function, and prevent further damage to the joint. Here are some key points regarding the management of hip impingement: Conservative Management may include: Rest and activity modification; Physical therapy; Medications: Nonsteroidal anti-inflammatory drugs (NSAIDs); Injections; Assistive devices. Surgical Intervention include: Hip arthroscopy; Open hip surgery; Osteotomy. It's important to note that early detection and timely intervention can lead to better outcomes. While hip impingement cannot be fully cured, appropriate treatment can significantly reduce pain, improve hip function, and prevent further joint damage. Consulting with an orthopedic specialist is crucial to determine the most suitable treatment approach based on your specific situation.

# **2. questionnaire survey**

**Survey on the Quality of ChatGPT Responses to Femoroacetabular Impingement (FAI) Related Questions**

**Question 1** What is your clinical title? [Single choice question]

| options | Subtotal | proportions |
| --- | --- | --- |
| resident physician | 4 | 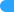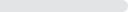10% |
| deputy chief physician | 23 | 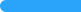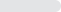57.5% |
| chief physician | 13 | 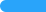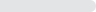32.5% |
| Number of valid entries for this question | 40 |  |

**Question 2** What is your annual volume of hip arthroscopy procedures? [Single choice question]

| options | Subtotal | proportions |
| --- | --- | --- |
| <10 units/year | 12 | 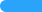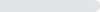30% |
| 10-20 units/year | 7 | 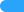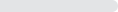17.5% |
| 20-50 units/year | 10 | 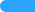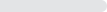25% |
| 50-100 units/year | 6 | 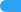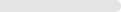15% |
| >100 units/year | 5 | 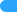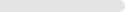12.5% |
| Number of valid entries for this question | 40 |  |

**Question 3** How many years have you been practicing hip arthroscopy? [Single choice question]

| options | Subtotal | proportions |
| --- | --- | --- |
| Within 1 year | 3 | 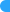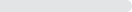7.5% |
| 1-3 years | 8 | 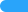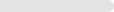20% |
| 3-5 years | 8 | 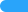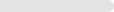20% |
| 5-10 years | 12 | 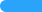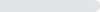30% |
| Greater than 10 years | 9 | 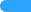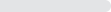22.5% |
| Number of valid entries for this question | 40 |  |

**Please evaluate the quality of ChatGPT's answers to questions concerning Femoroacetabular Impingement Syndrome based on your expertise. In your evaluation, consider the accuracy, completeness, relevance, and understandability of the responses. Select from the following options:**

1. Very Satisfied - The answers are accurate, detailed, and fully meet medical professional standards.

2. Satisfactory - The answers are generally accurate with comprehensive information.

3. General - The answers are correct; however, some information is incomplete or requires correction.

4. Unsatisfactory - The answers contain multiple inaccuracies and lack essential details.

5. Very Dissatisfied - The answers are incorrect or misleading and of poor informational quality.

**Question 4**

**Ask:** How do you fix femoroacetabular impingement?

**ChatGPT Response:** Femoroacetabular impingement (FAI) can be managed and treated through both conservative (non-surgical) and surgical approaches. The appropriate treatment depends on the severity of symptoms, the underlying cause, and the individual's specific circumstances. Here are some common treatment options for femoroacetabular impingement:Conservative Treatment(Rest and activity modification and Physical therapy，Nonsteroidal anti-inflammatory drugs ,Injections) and Surgical Treatment(Hip arthroscopy,Open hip surgery ,Osteotomy).The choice of treatment depends on various factors, such as the patient's age, activity level, severity of symptoms, and any associated hip conditions. It is important to consult with a qualified orthopedic specialist who can evaluate your specific situation and recommend the most appropriate treatment option for you.

**Evaluation Request:** What do you think of the quality of ChatGPT’s answer to this question? [Matrix radio question].

| Title \Option | Very Dissatisfied | Unsatisfactory | General | Satisfactory | Very Satisfied |
| --- | --- | --- | --- | --- | --- |
| Is the information in the answer medically accurate? | 2 (5%) | 0 (0%) | 11 (27.5%) | 19 (47.5%) | 8 (20%) |
| Could the answer potentially mislead patients, thereby posing health risks? | 1 (2.5%) | 1 (2.5%) | 13 (32.5%) | 15 (37.5%) | 10 (25%) |
| Is the information provided suitable for actual patient use? | 1 (2.5%) | 2 (5%) | 12 (30%) | 18 (45%) | 7 (17.5%) |
| Does the answer comprehensively cover the relevant information that users may need? | 1 (2.5%) | 1 (2.5%) | 13 (32.5%) | 20 (50%) | 5 (12.5%) |

**Question 5**

**Ask:** What causes hip impingement syndrome?

**ChatGPT Response:** Hip impingement syndrome, also known as femoroacetabular impingement (FAI), There are two main types of FAI: cam impingement and pincer impingement, and in some cases, both types can be present simultaneously. Here's a breakdown of the causes for each type of impingement:Cam Impingement(Excess bone growth,Abnormal hip joint mechanics)and Pincer Impingement(Overcoverage of the acetabulum,Abnormal hip socket alignment).Consulting with an orthopedic specialist can provide a more accurate.

**Evaluation Request:** What do you think of the quality of ChatGPT’s answer to this question? [Matrix radio question].

| Title \Option | Very Dissatisfied | Unsatisfactory | General | Satisfactory | Very Satisfied |
| --- | --- | --- | --- | --- | --- |
| Is the information in the answer medically accurate? | 1 (2.5%) | 2 (5%) | 16 (40%) | 14 (35%) | 7 (17.5%) |
| Is the answer likely to mislead patients and lead to health risks? | 1 (2.5%) | 4 (10%) | 14 (35%) | 13 (32.5%) | 8 (20%) |
| Is the information provided appropriate for actual patient use? | 1 (2.5%) | 3 (7.5%) | 13 (32.5%) | 17 (42.5%) | 6 (15%) |
| Does the answer comprehensively cover the relevant information that users may need? | 1 (2.5%) | 5 (12.5%) | 14 (35%) | 14 (35%) | 6 (15%) |

**Question 6**

**Ask:** What is hip femoroacetabular impingement syndrome?

**ChatGPT Response:** Femoroacetabular impingement syndrome is a condition characterized by abnormal contact between the femoral hea and the acetabulum in the hip joint. FAI syndrome can be categorized into two main types: 1.Cam Impingement; 2.Pincer Impingement. The abnormal contact can cause inflammation, pain, labral tears and eventual cartilage damage or hip osteoarthritis if left untreated. Symptoms may include hip pain, stiffness, clicking or catching sensations, limited range of motion, and discomfort during activities like walking, sitting, or sports. Diagnosis typically involves a combination of medical history assessment, physical examination, imaging studies and sometimes diagnostic hip injections to confirm the source of pain. Treatment for FAI syndrome can involve conservative measures and surgical measures. It's important to consult with a qualified orthopedic specialist to evaluate your symptoms.

**Evaluation Request:** What do you think of the quality of ChatGPT’s answer to this question? [Matrix radio question].

| Title \Option | Very Dissatisfied | Unsatisfactory | General | Satisfactory | Very Satisfied |
| --- | --- | --- | --- | --- | --- |
| Is the information in the answer medically accurate? | 1 (2.5%) | 2 (5%) | 10 (25%) | 19 (47.5%) | 8 (20%) |
| Is the answer likely to mislead patients and lead to health risks? | 1 (2.5%) | 1 (2.5%) | 15 (37.5%) | 16 (40%) | 7 (17.5%) |
| Is the information provided appropriate for actual patient use? | 1 (2.5%) | 2 (5%) | 7 (17.5%) | 23 (57.5%) | 7 (17.5%) |
| Does the answer comprehensively cover the relevant information that users may need? | 2 (5%) | 2 (5%) | 10 (25%) | 18 (45%) | 8 (20%) |

**Question 7**

**Ask:** Can you fix hip impingement without surgery?

**ChatGPT Response:** In some cases, femoroacetabular impingement (FAI), can be managed without surgery, especially if the symptoms are not severe. Here are some non-surgical options for managing hip impingement: 1.Physical Therapy: Using exercise program to strengthen the hip muscles. 2.Activity Modification: Avoid or modify activities that exacerbate symptoms. 3.Medications: Nonsteroidal anti-inflammatory drugs (NSAIDs), may be recommended to reduce pain and inflammation. 4.Injections: Corticosteroid injections into the hip joint can provide temporary relief by reducing inflammation and pain. 5. Assistive Devices: In some cases, using crutches or a cane to offload the hip joint during weight-bearing activities can help reduce symptoms and allow the joint to heal. If conservative treatments do not sufficiently alleviate symptoms or if there is significant joint damage, surgical intervention may be recommended.

**Evaluation Request:** What do you think of the quality of ChatGPT’s answer to this question? [Matrix radio question].

| Title \Option | Very Dissatisfied | Unsatisfactory | General | Satisfactory | Very Satisfied |
| --- | --- | --- | --- | --- | --- |
| Is the information in the answer medically accurate? | 1 (2.5%) | 4 (10%) | 10 (25%) | 16 (40%) | 9 (22.5%) |
| Is the answer likely to mislead patients and lead to health risks? | 1 (2.5%) | 6 (15%) | 13 (32.5%) | 14 (35%) | 6 (15%) |
| Is the information provided appropriate for actual patient use? | 1 (2.5%) | 5 (12.5%) | 9 (22.5%) | 16 (40%) | 9 (22.5%) |
| Does the answer comprehensively cover the relevant information that users may need? | 1 (2.5%) | 4 (10%) | 13 (32.5%) | 15 (37.5%) | 7 (17.5%) |

**Question 8**

**Ask:** Can hip impingement be cured?

**ChatGPT Response:** Femoroacetabular impingement (FAI), is a structural abnormality in the hip joint. While it cannot be completely "cured" in the sense of reversing the underlying anatomy, it can often be effectively managed and treated. The goal of treatment is to reduce symptoms, improve hip function, and prevent further damage to the joint. Here are some key points regarding the management of hip impingement: Conservative Management may include: Rest and activity modification; Physical therapy; Medications: Nonsteroidal anti-inflammatory drugs (NSAIDs); Injections; Assistive devices. Surgical Intervention include: Hip arthroscopy; Open hip surgery; Osteotomy. It's important to note that early detection and timely intervention can lead to better outcomes. While hip impingement cannot be fully cured, appropriate treatment can significantly reduce pain, improve hip function, and prevent further joint damage. Consulting with an orthopedic specialist is crucial to determine the most suitable treatment approach based on your specific situation.

**Evaluation Request:** What do you think of the quality of ChatGPT’s answer to this question? [Matrix radio question].

| Title \Option | Very Dissatisfied | Unsatisfactory | General | Satisfactory | Very Satisfied |
| --- | --- | --- | --- | --- | --- |
| Is the information in the answer medically accurate? | 1 (2.5%) | 2 (5%) | 10 (25%) | 19 (47.5%) | 8 (20%) |
| Is the answer likely to mislead patients and lead to health risks? | 1 (2.5%) | 2 (5%) | 11 (27.5%) | 18 (45%) | 8 (20%) |
| Is the information provided appropriate for actual patient use? | 1 (2.5%) | 3 (7.5%) | 8 (20%) | 21 (52.5%) | 7 (17.5%) |
| Does the answer comprehensively cover the relevant information that users may need? | 1 (2.5%) | 3 (7.5%) | 10 (25%) | 19 (47.5%) | 7 (17.5%) |

**Question 9**

Based on your evaluation of ChatGPT's responses to questions regarding femoroacetabular impingement syndrome (FAI), do you believe that ChatGPT could serve as a reliable healthcare resource for patients seeking initial medical information? Please assess its accuracy, completeness, applicability, and safety. [Multiple choice question].

| options | Subtotal | proportions |
| --- | --- | --- |
| Very Well Equipped - Fully able to fulfill the required conditions. | 5 | 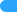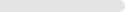12.5% |
| Able - Adequately meets the conditions. | 20 | 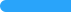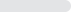50% |
| Neutral - Neither fully meets nor fails to meet the conditions. | 11 | 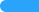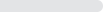27.5% |
| Insufficient - Does not fully meet the required conditions. | 3 | 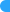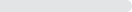7.5% |
| Very Short Of - Significantly lacks the necessary resources. | 1 | 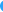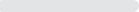2.5% |
| Number of valid entries for this question | 40 |  |
